# Kintsugi, Mending with Gold - A Psychotherapeutic Technique

**DOI:** 10.1192/j.eurpsy.2025.2155

**Published:** 2025-08-26

**Authors:** P. I. Santos, A. B. Costa

**Affiliations:** 1 Hospital das Neurociências, Hospital Trofa Saúde, Vila do Conde; 2 HPG, VNG; 3 Psichology, Psicobodycare, Porto, Portugal

## Abstract

**Introduction:**

Psychotherapy, when supported by an appropriate set of techniques, has a high impact on the patient’s psychological well-being, self-knowledge and self-growth.

**Objectives:**

This study aimed to determine the clinical utility of the therapeutic tool “Kintsugi,” developed by the corresponding author, through two assessment phases.

**Methods:**

To a total of 200 participants, aged between 18 and 70 years (M = 44.3, SD = 12.5), were administered a semistructured interview, the Hamilton Anxiety Scale, and the Échelle de Mesure des Manifestations du Bien-Être Psychologique. The assessments occurred before and after the application of the techinique, with an interval of about two months (approximately 4 to 6 sessions).

**Results:**

Findings indicate that, in the first moment of assessment, participants showed lower levels of psychological well-being (M = 78.0, SD = 15.0) and, consequently, more anxious symptoms (M = 32.0, SD = 9.0). After the use of the “Kintsugi” technique, regardless of the participant’s age or gender, an increase in psychological well-being (M = 96.5, SD = 12.0; F-test = 77.65, p < .001, η² = 0.28) and a decrease in anxiety (M = 21.0, SD = 7.0; F-test = 87.50, p < .001, η² = 0.31) were observed.

**Conclusions:**

This therapeutic tool proves to be useful, beneficial, and clinically effective in a psychotherapeutic context, as its high contribution to the mental health of individuals becomes evident.

**Disclosure of Interest:**

None Declared

